# Exercise-Induced ECG Abnormalities in Pediatric Pectus Excavatum: Evidence of Right Ventricular Compression Beyond the Haller Index

**DOI:** 10.3390/medsci14030379

**Published:** 2026-07-08

**Authors:** Karine Guerrier, Aram Bejnood, Sharvari Shyam, Rebecca Ann Hyde, Benjamin Hendrickson, Trey Eubanks, Tim Jancelewicz, Ranjit Philip

**Affiliations:** 1 Department of Pediatric Cardiology, Le Bonheur Children’s Hospital, Memphis, TN 38103, USA; kguerrie@uthsc.edu (K.G.); ann.hyde@lebonheur.org (R.A.H.); bhendri8@uthsc.edu (B.H.); rphilip@uthsc.edu (R.P.); 2 Department of Pediatric Cardiology, The University of Tennessee Health Science Center, Memphis, TN 38163, USA; 3 Department of Internal Medicine, The University of Tennessee Health Science Center, Memphis, TN 38163, USA; abejnoo1@uthsc.edu; 4 Department of Pediatric Surgery, Le Bonheur Children’s Hospital, Memphis, TN 38103, USA; jeubank1@uthsc.edu (T.E.); tjancele@uthsc.edu (T.J.); 5 Department of Pediatric Surgery, The University of Tennessee Health Science Center, Memphis, TN 38163, USA

**Keywords:** Haller index, pectus excavatum, right ventricular compression, premature ventricular complexes

## Abstract

Background: Pectus excavatum (PEX) is the most common congenital chest wall deformity and may result in cardiac compression and arrhythmias. The relationship between structural severity and exercise-induced electrocardiographic (ECG) abnormalities in pediatric patients remains unclear. Methods: We performed a retrospective study of patients aged 10–19 years that underwent standardized preoperative evaluation for PEX between 2015 and 2021, including ECG, transthoracic echocardiography (TTE), computed tomography (CT), and cardiopulmonary exercise testing (CPET). PEX severity was assessed using the Haller index (HI), while right ventricular (RV) compression was evaluated on CT. Tricuspid valve annular size (TVAS) on TTE was used as a surrogate marker of RV compression. Exercise-induced ECG abnormalities, including premature ventricular complexes (PVCs), were analyzed and correlated with HI, RV compression, and TVAS. Results: Among 124 patients (85% male; median age 15 years), 33% exhibited exercise-induced ECG abnormalities, most commonly PVCs (24% overall). PVC occurrence was not associated with Haller index severity (*p* = 0.35) but was significantly associated with RV compression on CT (92.6% vs. 62.1%, OR 7.64, *p* = 0.02). Patients with ECG abnormalities had significantly smaller TVAS compared to those without (1.98 ± 0.31 cm vs. 2.09 ± 0.33 cm, *p* = 0.04). Although PVCs were more frequent in patients with TVAS z-score ≤ −2.0, this did not reach statistical significance. Conclusions: Exercise-induced ventricular ectopy in pediatric PEX is associated with right ventricular compression rather than structural severity as defined by HI. Echocardiographic measures such as TVAS may serve as noninvasive markers of clinically significant compression. These findings highlight the importance of cardiac–thoracic relationships in predicting arrhythmic risk and suggest a potential for reversibility with surgical correction.

## 1. Introduction

Pectus excavatum (PEX) is a congenital chest wall deformity of the anterior wall of the thoracic cavity. It is the most common cause of pediatric chest wall deformities and disproportionately affects males more than females [1]. Posterior depression of the sternum and adjacent costal cartilage is associated with cardiac issues, particularly the right atrium and right ventricle (RV), as well as pulmonary compression. The degree of severity is determined by the Haller index (HI), based on the interior transverse chest diameter to anterior–posterior diameter ratio calculated on computed tomography scan [1]. In moderate to severe cases, with HI ≥ 3.2, compression may lead to impaired cardiac function, exercise intolerance, and tachyarrhythmia [2,3].

Atrial fibrillation, atrial tachycardia, and ventricular arrhythmia have been reported in adult and pediatric patients with PEX [4,5,6,7,8,9]. Cardiac rotation and mechanical compression of the right heart chambers have been postulated as potential etiologies for the arrhythmias [10]. At baseline, patients with PEX have been found to have characteristic electrocardiogram (ECG) findings including rSr’ and biphasic P waves with dominant negative force in lead V1 [11]. There have been case reports of Brugada phenocopy likely due to cardiac anatomical rotation and displacement that contribute to mechanical compression of the RV outflow tract [12,13]. While ECG findings of right bundle branch block, right atrial enlargement, right axis deviation, and ST-segment elevation on the right precordial leads can be seen at rest, the significance of exercise-induced ECG changes in pediatric patients with PEX has been of peak interest [14,15].

Recent large-scale studies have characterized the prevalence and severity of exercise-induced arrhythmias in pediatric PEX patients. Complex ventricular ectopy has been associated with higher structural severity indices including HI, correction index, and depression index [16]. Cardiac abnormalities have been reported in 70% of young PEX patients preoperatively, with RV compression present in 33.1%, all of which were resolved after surgical correction [17]. However, the relationship between structural severity and ECG abnormalities remains controversial, as another study found no association between HI or RV geometric distortion and ECG abnormalities in their cohort [18].

The purpose of this study was to determine the frequency and characteristics of exercise-induced ECG changes and arrhythmia in pediatric and adolescent patients with PEX. In addition, we evaluated whether ventricular ectopy frequency was related to structural severity of PEX or localized right ventricular compression based on deformity location.

We also explored whether tricuspid valve annular size (TVAS) could serve as an adjunctive echocardiographic marker of clinically significant right ventricular compression.

## 2. Materials and Methods

This study was conducted as a retrospective cohort analysis of pediatric and adolescent patients (ages 10–19 years) who underwent a standardized, institution-based preoperative evaluation for pectus excavatum (PEX) between January 2015 and December 2021. The study was approved by the Institutional Review Board of University of Tennessee Health Science Center (Protocol Code 22-08725-XP) on 19 July 2021. The institutional protocol included comprehensive cardiovascular and structural assessment using 12-lead ECG, transthoracic echocardiography (TTE), chest computed tomography (CT), and cardiopulmonary exercise testing (CPET).

### 2.1. Assessment of Pectus Severity and Cardiac Compression

PEX severity was quantified using the HI, calculated from CT imaging as the ratio of transverse chest diameter to the anterior–posterior chest diameter [19]. Consistent with established thresholds, mild PEX was defined as HI 2.0–3.19, while moderate and severe PEX were defined as HI ≥ 3.2, grouped together to align with what qualifies for PEX repair.

Right ventricular (RV) compression was assessed qualitatively on CT imaging by experienced readers, based on visual evidence of deformation of the RV free wall, reduction in RV chamber space, and displacement or rotation of cardiac structures. Because CT-based qualitative assessment can vary, an additional echocardiographic metric was incorporated. TVAS, obtained from TTE, was used as a surrogate marker of RV compression [20]. Measurements were indexed to body surface area and converted to z-scores based on Boston Children’s Hospital normative database incorporated within our echocardiography software, with a z-score ≤ −2.0 considered indicative of reduced annular dimension and potential RV compression.

### 2.2. Electrocardiographic and Exercise Testing Protocol

A standard resting 12-lead ECG was obtained for all participants prior to exercise testing, recorded at a paper speed of 25 mm/s and amplitude of 10 mm/mV. Continuous ECG monitoring was performed during CPET using a standardized Bruce protocol designed to achieve maximal exertion [3].

Electrocardiograms were systematically reviewed for both baseline and exercise-induced abnormalities. Specific findings evaluated included:

- Premature atrial complexes (PACs), with documentation of aberrant conduction;
- Premature ventricular complexes (PVCs);
- Ventricular tachycardia (VT);
- Repolarization abnormalities, including T-wave inversion > 1 mm in two or more contiguous leads [15,16].

Ectopic activity was further characterized by morphology (monomorphic vs. polymorphic), coupling interval, and complexity, defined as isolated beats, couplets, or triplets. The temporal relationship to exercise (early, peak, or recovery phase) was also noted when available.

### 2.3. Statistical Analysis

Exercise-induced ECG abnormalities were analyzed in relation to structural markers of PEX, including HI severity, qualitative RV compression on CT, and TVAS z-score on echocardiography. Categorical variables were compared using Pearson’s χ^2^ test, while continuous variables were analyzed using two-sample *t*-tests where appropriate.

Logistic regression analysis was performed to estimate odds ratios (ORs) for the association between ventricular ectopy and structural or functional measures of RV compression. A *p*-value < 0.05 was considered statistically significant. Statistical analyses were performed using standard statistical software.

## 3. Results

### 3.1. Baseline Characteristics

A total of 124 patients met inclusion criteria, with a strong male predominance (105 males, 85%). The median age of the cohort was 15 years (interquartile range 10–19 years). The majority of patients (85%) had moderate-to-severe PEX (HI ≥ 3.2), highlighting a cohort largely composed of patients referred for surgical evaluation (Table 1).

Right ventricular compression on CT imaging was identified in 79 patients (69% of those with available imaging), while reduced TVAS (z-score ≤ −2.0) was observed in 72 patients (58%), suggesting that structural cardiac impact was common within this population (Table 1).

### 3.2. Exercise-Induced ECG Abnormalities

Overall, 41 patients (33%) demonstrated exercise-induced ECG abnormalities during CPET (Table 1). Among these, PVCs were the most frequently observed abnormality, present in 30 patients (73% of those with ECG changes; 24% overall cohort). Less frequent abnormalities included PACs and non-sustained ventricular tachycardia, though these were not a major contributor to the overall event burden.

Premature ventricular contractions were predominantly isolated and monomorphic, with only a minority demonstrating increased complexity such as couplets or triplets. Most ectopy occurred during peak exercise or early recovery phases, consistent with hemodynamic and positional stress on the RV.

### 3.3. Association with Structural Markers

**Figure 1:**
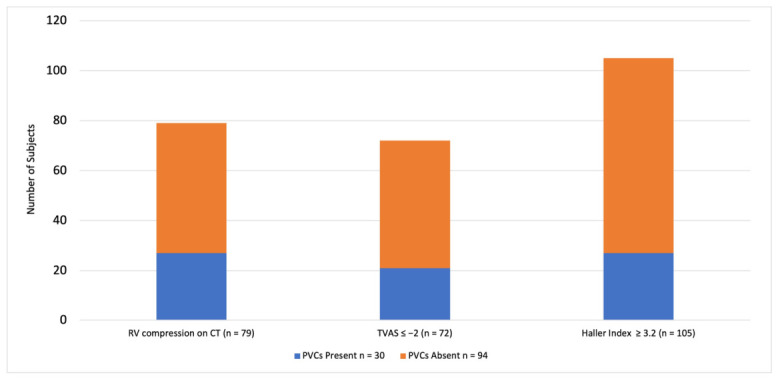
Association of Premature Ventricular Complexes with Right Ventricular Compression Assessed by CT, Echocardiography, and Haller Index. CT = computed tomography; PVC = premature ventricular complexes RV = right ventricle; TVAS = tricuspid valve annulus size.

When stratified by structural parameters, the prevalence of PVCs was higher in patients with CT-defined RV compression (34%) compared to those without compression. Similarly, PVCs were more frequent among patients with reduced TVAS (29%) vs. higher annular size, though this correlation did not reach statistical significance (Table 2, Figure 1).

Notably, there was no statistically significant association between PVC occurrence and PEX severity as measured by HI (χ^2^ = 0.86, *p* = 0.35). In contrast, a significant association was observed between PVCs and RV compression on CT imaging (χ^2^ = 5.20, *p* = 0.02).

**Figure 2:**
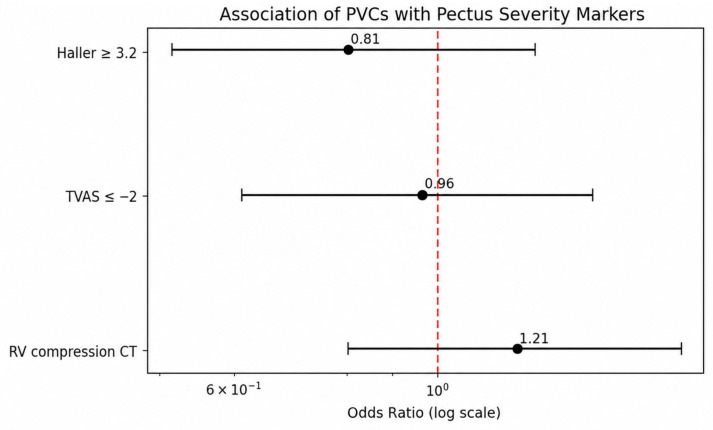
Forest plot showing association of PVCs with pectus excavatum severity markers (RV compression on CT, TVAS z-score ≤ −2, and Haller index ≥ 3.2). The red dashed vertical line is the null line, representing an odds ratio (OR) of 1.0. Odds ratios with 95% CIs are displayed on a log scale; RV compression was significantly associated with PVCs, while other markers were not. CT = computed tomography; PVC = premature ventricular complexes; RV = right ventricle; TVAS = tricuspid valve annulus size.

Among patients with PVCs, 92.6% demonstrated evidence of RV compression, corresponding to a markedly increased likelihood of ectopy (OR 7.64, 95% CI 1.70–34.37; *p* = 0.02; Figure 2).

### 3.4. Echocardiographic Correlates

Mean TVAS was significantly smaller in patients with exercise-induced ECG abnormalities compared with those without abnormalities (1.98 ± 0.31 cm vs. 2.09 ± 0.33 cm, *p* = 0.04), suggesting that reduced tricuspid annular dimension may reflect clinically meaningful cardiac compression.

Although a TVAS z-score ≤ −2.0 was more common among patients with PVCs, this association did not reach statistical significance (χ^2^ = 0.52, *p* = 0.46). However, among patients with CT-confirmed RV compression, 52% also demonstrated a reduced TVAS z-score, supporting concordance between imaging modalities.

Taken together, these findings demonstrate that exercise-induced ventricular ectopy is relatively common in pediatric PEX patients. RV compression, rather than global deformity severity (HI), appears to be the primary structural correlate of arrhythmia risk.

## 4. Discussion

In this cohort of pediatric and adolescent patients with PEX, exercise-induced ECG abnormalities were observed in approximately one-third of individuals, with PVCs representing the most frequent finding. The most important insight from this study is that the occurrence of ventricular ectopy appears to be more closely associated with localized right ventricular compression than with traditional measures of global chest wall deformity severity, such as the HI.

The observed prevalence of exercise-induced PVCs (24%) underscores that ventricular ectopy is relatively common in this population. Compared with the 34.9% prevalence reported by Piczer et al., our cohort demonstrated a somewhat lower frequency; however, differences in study design, patient selection, and definitions of arrhythmia burden likely contribute to this variation [16]. Importantly, this finding has practical implications for clinicians—including pediatric cardiologists, pediatricians, and anesthesiologists—as the presence of this degree of ectopy should not delay clearance for PEX repair. Rather, the ectopy appears to be a physiologic consequence of the underlying chest wall deformity, with evidence suggesting improvement or resolution following surgical correction [11,21,22].

Prior work has emphasized the relationship between complex ventricular ectopy and increasing structural severity, whereas our study focused primarily on the presence of ectopy [16]. This distinction is critical. It is plausible that structural severity indices such as HI correlate more strongly with the burden, morphology, and complexity of arrhythmias, rather than their mere occurrence. This nuance may help reconcile inconsistencies across studies and highlights the importance of more granular arrhythmia characterization in future investigations.

*Revisiting the Role of the Haller Index*

Our findings add to a growing body of literature questioning the utility of HI as a standalone metric for predicting cardiac involvement in PEX [18,23,24,25,26]. Although HI remains a widely used and objective measure of thoracic deformity, it is fundamentally a two-dimensional geometric index that does not adequately account for cardiac displacement and rotation, asymmetry of sternal depression, localized compression of cardiac chambers and dynamic changes during respiration and exercise.

The lack of association between HI and exercise-induced PVCs in this study suggests that global deformity severity does not necessarily equate to physiologic or electrophysiologic consequence. This aligns with findings by Kohli et al. and Lukács et al., both of whom demonstrated that cardiac abnormalities may occur independently of HI severity [17,18]. Kohli et al. found no association between HI and ECG abnormalities in their cohort of 28 patients, despite 60% having abnormal ECGs and 86% having RV geometric distortion [18]. Lukács et al. reported that while pectus excavatum severity correlated with the presence of any cardiac abnormality, it did not correlate with individual abnormality types [17].

Taken together, these data support a paradigm shift away from reliance on HI alone toward a more integrated, physiology-informed assessment. This has direct clinical and policy implications, particularly in the context of insurance approval for PEX repair, which is often predicated on a threshold HI value (e.g., ≥3.2). Such an approach risks overlooking patients with significant physiologic or electrophysiologic compromise despite “non-severe” structural indices. A more comprehensive evaluation that incorporates factors such as deformation location, degree of cardiac compression, and functional/electrocardiographic impact is therefore essential to accurately assess disease burden and guide appropriate intervention.

*Mechanistic Basis of Ventricular Ectopy*

The strong association between PVCs and CT-defined RV compression supports a mechanistic model in which mechanical and hemodynamic perturbations directly influence arrhythmogenesis. Several interrelated mechanisms are likely to contribute [27]. Mechanical deformation of the RV free wall and RV outflow tract can lead to impaired RV filling and preload limitation. Chronic compression may alter myocardial fiber orientation and stretch-sensitive ion channel activity, predisposing to ectopic depolarization. Dynamic cardiac rotation and displacement, relative ischemia and reduced oxygen delivery, decreased maximal oxygen consumption and limited cardiac output reserve may contribute to a substrate for ventricular ectopy. Reduced diastolic filling may create heterogeneous myocardial stress and electrophysiologic instability during exertion. Exercise amplifies respiratory effort and intrathoracic pressure changes, which may exaggerate cardiac positional shifts and transiently worsen RV compression [24,25]. Sympathetic activation may further lower arrhythmic thresholds in the setting of structurally compromised myocardium.

The observation that over 90% of patients with PVCs had CT evidence of RV compression strongly reinforces the central role of localized mechanical factors. Notably, these mechanisms are likely dynamic and exercise-dependent, which explains why abnormalities may not be evident on resting ECG alone.

Chan Wah Hak et al. reported a case of exercise-induced ventricular tachycardia in a 19-year-old with PEX that resolved completely after surgical correction, with no recurrence at 3-year follow-up, further supporting the proposed mechanical etiology [11].

*Integration of Echocardiographic Findings*

Due to posterior depression of the anterior thoracic wall, right atrial and right ventricular compression are not uncommon in patients with PEX, particularly given the anterior position of the right ventricle. However, TTE assessment of the right heart is often limited by poor acoustic windows related to the musculoskeletal deformity and interposed lung artifact, making visualization of right-sided structures challenging. As a result, the degree of right heart compression may be subjectively underestimated on TTE. Lain et al. [26] reported underestimation of right heart compression in up to 70% of their cohort using transthoracic echocardiography, whereas transesophageal echocardiography demonstrated right heart compression and distortion of the tricuspid valve annulus in all subjects. These limitations highlight the need for objective TTE markers, such as TVAS, to better capture subtle right heart constraint and reduce reliance on subjective interpretation in the assessment of RV compression in PEX [27,28].

The incorporation of TVAS as a surrogate marker of RV compression provides an important complementary perspective. The finding that patients with exercise-induced ECG abnormalities had significantly smaller TVAS measurements suggests that annular restriction reflects underlying RV constraint.

From a clinical standpoint, this is particularly valuable because echocardiography is widely available and non-invasive. However, the incomplete correlation between TVAS and CT-defined compression highlights an important limitation: TVAS is not sufficiently sensitive to detect all cases of RV compression identified by advanced imaging. This discrepancy likely reflects variation in the location and geometry of pectus-related compression, particularly in cases with complex three-dimensional distortion or posterior displacement that may not be adequately captured by standard echocardiographic windows.

Thus, TVAS should be viewed as a screening or adjunctive marker rather than a definitive measure of RV compression. While a reduced TVAS can be helpful in identifying the presence of RV constraint—even in patients without severe HI—a normal TVAS does not exclude clinically meaningful compression. Additionally, TVAS provides an objective, reproducible marker for the imager to assess RV compression, reducing reliance on subjective interpretation and helping avoid the common tendency to label studies as “normal” despite subtle evidence of right heart constraint. Smaller TVAS is thought to reflect a greater degree of right ventricular compression [27,28]. Accordingly, TVAS on may serve as a useful marker to identify patients at increased risk for exercise-induced rhythm disturbances. While this does not obviate the need for CT, it may help reduce the frequency of radiation exposure in selected cohorts.

Whether relief of right heart compression and subsequent normalization of tricuspid valve annular size following surgical intervention lead to resolution of exercise-induced electrocardiographic changes and ventricular ectopy warrants further investigation in larger, prospective cohorts.

These findings further reinforce that the physiologic and electrophysiologic impact of PEX depends not only on the degree of deformity but also on its location and interaction with cardiac structures, underscoring the need for a more comprehensive, multimodality approach to assessment.

This study has several practical implications for the evaluation of pediatric PEX. HI alone is insufficient to assess cardiac risk or arrhythmogenic potential. Functional testing, including cardiopulmonary exercise testing (CPET), can uncover clinically relevant abnormalities that are not evident at rest. Multimodality imaging (CT in conjunction with echocardiography) enhances detection of physiologically significant cardiac compression. Collectively, these findings support a more comprehensive and individualized evaluation strategy, particularly in patients presenting with symptoms such as exercise intolerance, palpitations, or syncope despite a HI < 3.2.

These results further support the development of a formalized cardiovascular evaluation pathway for patients with PEX, incorporating baseline electrocardiography, CPET with rhythm monitoring, transthoracic echocardiography with TVAS measurement, and selective use of CT or cardiac magnetic resonance imaging (MRI), analogous to other algorithm-based clinical assessment strategies [29,30].

Given the limitations of both HI and echocardiography, cardiac MRI represents a promising modality for future evaluation. MRI offers several advantages, including three-dimensional assessment of thoracic and cardiac anatomy, quantification of right ventricular volumes and function, evaluation of myocardial strain and flow dynamics, and—importantly—the absence of ionizing radiation. However, barriers such as cost, limited accessibility, and longer acquisition times currently restrict its widespread implementation. Nonetheless, MRI may play a particularly valuable role in borderline cases or in patients with discordant findings across imaging modalities.

*Limitations*

This study is limited by its retrospective design and the absence of a control group. Arrhythmia assessment was confined to the exercise testing environment, and the lack of ambulatory monitoring limits conclusions regarding overall arrhythmia burden. Additionally, echocardiographic measurements were interpreted by a single pediatric cardiologist, whereas CT studies were interpreted by multiple pediatric radiologists as part of routine clinical care. Although this reflects real-world practice, interobserver variability in CT assessment of RV compression may have influenced our findings. Finally, because this was a retrospective study, all eligible patients meeting inclusion criteria during the study period were included, and no formal a priori sample size calculation was performed.

*Clinical Implications and Future Directions*

Our findings have potential implications for the clinical evaluation of pediatric patients with PEX. Based on these observations, we suggest an initial evaluation consisting of clinical assessment, ECG, TTE, CPET, and assessment of pectus severity using the HI. Patients demonstrating exercise-induced ECG abnormalities, reduced TVAS, or evidence of RV compression may benefit from multidisciplinary evaluation for potential surgical intervention. CT imaging remains an essential component of preoperative planning and Nuss bar sizing and is therefore typically reserved closer to the time of surgical repair. Although not evaluated in the present study, cardiac MRI has the potential to provide comprehensive structural and functional assessment without ionizing radiation and may further refine risk stratification in selected patients.

Future prospective studies should validate this proposed multimodality approach, including longitudinal assessment before and after surgical repair to determine whether relief of right ventricular compression reduces ventricular ectopy. Advanced imaging modalities, such as cardiac MRI, along with further evaluation of exercise physiology and autonomic function, may improve risk stratification and provide additional insight into the mechanisms of arrhythmogenesis in this population.

Ultimately, a more nuanced understanding of the relationship between structure, function, and electrophysiology in PEX will allow for more precise identification of patients who may benefit from closer monitoring or earlier intervention.

## 5. Conclusions

Exercise-induced ventricular ectopy in pediatric PEX appears to be more closely associated with RV compression than with structural severity as defined by HI. Echocardiographic parameters such as TVAS may serve as useful, noninvasive markers of clinically significant cardiac compression. These findings highlight that the anatomic relationship of the deformity to the heart, rather than severity indices alone, may better predict arrhythmic risk.

## Figures and Tables

**Table 1:** Baseline Demographic and Clinical Characteristics of the Study Population.

Variable	Overall Cohort (*n* = 124)
Median age in years	15 (10–19)
Male sex	105 (85%)
Exercise-induced ECG changes	41 (33%)
PVCs (%)	30 (24%)
RV compression on CT	79 (69%) (*n* = 114)
Haller Index ≥ 3.2	105 (85%)
TVAS ≤ −2	72 (58%)
pediatric patients	

CT = computed tomography; PVC = premature ventricular complexes; RV = right ventricle; TVAS = tricuspid valve annulus size.

**Table 2:** Association of Premature Ventricular Complexes with Right Ventricular Compression Assessed by CT, Echocardiography, and Haller Index.

Variable	PVCs Present	PVCs Absent	*p*-Value
RV compression CT (*n* = 79)	27 (34%)	52 (66%)	0.02
TVAS ≤ −2 (*n* = 72)	21 (29%)	51 (71%)	0.46
Haller Index ≥ 3.2 (*n* = 105)	27 (26%)	78 (74%)	0.35

CT = computed tomography; PVC = premature ventricular complex; RV = right ventricle; TVAS = tricuspid valve annulus size.

## Data Availability

The original contributions presented in this study are included in the article. Further inquiries can be directed to the corresponding author.

## Abbreviations

The following abbreviations are used in this manuscript:

PEX	Pectus Excavatum
CT	Computed Tomography
TTE	Transthoracic Echocardiogram
ECG	Electrocardiogram
TVAS	Tricuspid Valve Annulus Size
PVC	Premature Ventricular Complexes
HI	Haller Index
RV	Right Ventricle
